# An interpretative phenomenological analysis (IPA) of coercion towards community dwelling older adults with dementia: findings from Mysore studies of natal effects on ageing and health (MYNAH)

**DOI:** 10.1007/s00127-016-1286-4

**Published:** 2016-09-29

**Authors:** Vijay Danivas, Mufaddal Bharmal, Paul Keenan, Steven Jones, Samuel Christaprasad Karat, Kumaran Kalyanaraman, Martin Prince, Caroline H. D. Fall, Murali Krishna

**Affiliations:** 1 Foundation for Research Advocacy in Mental Health (FRAMe), Mysore, India; 2 Northwestern Mental Health, Melbourne, Australia; 3 Medical School, University of Southampton, Southampton, UK; 4 Edge Hill University, Lancashire, UK; 5 CSI Holdsworth Memorial Hospital, Mysore, India; 6 Psychiatric Epidemiology, Institute of Psychiatry, London, UK; 7 International Pediatric Epidemiology, MRC Lifecourse Epidemiology Unit, University of Southampton, Southampton, UK; 8 Wellcome DBT India Alliance at Epidemiology Research Unit, CSI Holdsworth Memorial Hospital, Mysore, India; 9 Broadmeadows Health Centre, 35 Johnstone Street, Broadmeadows, VIC 3033 Australia

**Keywords:** Dementia, Coercion, Caregiving, Low- and middle-income setting, Narrative methods, Interpretative phenomenological analysis

## Abstract

**Purpose:**

Limited availability of specialist services places a considerable burden on caregivers of Persons with Dementia (PwD) in Low- and Middle-Income Countries (LMICs). There are limited qualitative data on coercive behavior towards PwD in an LMIC setting.

**Aim:**

The aim of this study was to find relevant themes of the lived experience of relatives as caregivers for PwD in view of their use of coercive measures in community setting in South India.

**Method:**

Primary caregivers (*n* = 13) of PwDs from the Mysore study of Natal effects on Ageing and Health (MYNAH) in South India were interviewed to explore the nature and impact of coercion towards community dwelling older adults with dementia. The narrative data were coded using an Interpretative Phenomenological Analysis (IPA) approach for thematic analysis and theory formation.

**Results:**

Caregivers reported feeling physical and emotional burn-out, a lack of respite care, an absence of shared caregiving arrangements, limited knowledge of dementia, and a complete lack of community support services. They reported restrictions on their lives through not being able take employment, a poor social life, reduced income and job opportunities, and restricted movement that impacted on their physical and emotional well-being. Inappropriate use of sedatives, seclusion and environmental restraint, and restricted dietary intake, access to finances and participation in social events, was commonly reported methods of coercion used by caregivers towards PwD. Reasons given by caregivers for employing these coercive measures included safeguarding of the PwD and for the management of behavioral problems and physical health.

**Conclusion:**

There is an urgent need for training health and social care professionals to better understand the use of coercive measures and their impact on persons with dementia in India. It is feasible to conduct qualitative research using IPA in South India.

## Background

Neurocognitive disorders are a major cause of disability and mortality in the late life and are associated with high costs for health systems and society particularly in low- and middle-income countries (LMIC), such as India [1–3]. Population-based studies in India report prevalence rates of 7.5 and 10.6% for dementia in those aged above 60 years in urban and rural areas, respectively [2, 4]. The proportion of persons with dementia in India is expected to increase twofold by 2030 because of the steady growth in the older population and stable increments in life expectancy [3]. Dementia is a major cause of disability and this has a disproportionate impact on capacity for independent living in later life [1–3, 5, 6].

In India, the characteristics of dementia are considered non-pathological and a part of normal ageing [7, 8]. The psychological and behavioral problems seen in dementia are associated with stigma, and this can lead to neglect and sometimes abuse of the elderly [9].

Formal care arrangements for Indian elders in the public health sector are sparse [9–11]. The specialties of old age psychiatry or elderly medicine are poorly established, and there is virtually no facilities of continuing care to meet the complex medical and psychosocial needs of persons with dementia (PwD) and their families. In addition, the number of residential places for elders with conditions, such as dementia, is very low. Care for those with high-dependency needs is almost entirely family based with very limited formal services. Thus, the family remains the primary source of care and supports for the vast majority of PwD in India [9, 11, 12].

Caring for PwD is associated with a greater physical, mental, and financial burden on the caregiver [1, 7]. A small number of studies in India have examined the impact of care giving for elders [1, 3]. Care givers of PwD spend significant periods of time providing care than many other longer term conditions, including communicating, supervising, and helping with daily living tasks, e.g., eating and toileting [12, 13]. Caregivers are at increased risk of having a common mental disorder [14] and subject to significant economic strain, because a high proportion leaves employment to provide care due to high health care costs [13]. In addition, the disease course and progressive care burden for the PwD often lead to carers adopting increased direct care roles.

With disease progression, PwDs may lose the capacity to make important decisions, resulting in caregivers having to make these decisions in their best interests [15]. Coercion is defined as “the action or practice of persuading someone to do something by using force or threat” [16]. However, inadvertent coercive measures can lead to neglect and poor clinical outcomes with medicolegal complications [17, 18].

To our knowledge, there has been no study in an LMIC setting, including India that has examined why caregivers use coercive measures towards PwDs in the community. Understanding coercive practices will inform the development of training and policies aimed at safeguarding both PwDs and their caregivers. With this background, we carried out an exploratory study by interviewing primary caregivers of PwD from the ongoing Mysore studies on Natal effects on Ageing and Health (MYNAH) in South India [19]. The aim was to identify methods of coercion and explore the caregivers’ reasons for using them.

## Methods

### Setting

This study was carried out at the Epidemiology Research Unit, CSI Holdsworth Memorial Hospital (HMH), Mysore, South India. The study was approved by HMH Research and Ethics Committee.

### Recruitment

Between 1993 and 2001, 3427 men and women born during 1934–1966 at HMH were located by a house-to-house survey of the area of surrounding HMH, and matched to their birth records. They constitute the Mysore Birth Records Cohort. Surviving members of this cohort were asked to participate in a follow-up study to measure cognitive function, cardiometabolic disorders, and mental disorders in the late life. Between March 2013 and March 2014, we examined 428 men and women from this cohort aged 55–80 years for cardiometabolic disorders and mental disorders, including dementia. Table 1 provides a list of investigations and assessments conducted for deriving a 10/66 diagnosis of dementia (see Table 1). Cognitive functioning as a continuous measure was obtained by administering the 10/66 battery of cognitive tests; Dementia was defined by a score above a cut-off point of predicted probability of DSM IV Dementia Syndrome from the logistic regression equation of the 10/66 dementia diagnostic algorithm [20].

**Table 1:** Assessments for deriving a 10/66 diagnosis of dementia

Battery of Cognitive tests	(1) The Community Screening Instrument for Dementia (CSI’D’) COGSCORE incorporating the CERAD animal naming verbal fluency task. (CERAD-Consortium to Establish a register for Alzheimer’s Disease) (2) The modified CERAD 10 word list learning task with delayed recall (3) Informant interview, the CSI’D’ RELSCORE, for evidence of cognitive and functional decline
Instruments for diagnosis of dementia	(1) Battery of cognitive tests (listed above) (2) A structured clinical mental state interview, the Geriatric Mental State, which applies a computer algorithm (3) An extended informant interview, the History and Aetiology Schedule-Dementia Diagnosis and subtype (4) The NEUROEX, a brief fully structured neurological assessment (5) Behavioral and Psychological symptoms: assessed by Neuropsychiatric Inventory

Of the 428, 14 (8 men and 6 women) were diagnosed with dementia and their primary caregivers were invited to participate in this study. Of the 14, 13 participants (7 men and 6 women), caregivers were recruited. One caregiver was willing to participate, but was not living in Mysore. The average age of the PwD was 73 years. The primary caregivers were 8 sons, 4 daughters, and 1 daughter-in-law, aged 28–63 years. Of the 13 caregivers, 5 were employed, 3 were retired, and 5 were involved in household responsibilities. Education levels ranged from no formal education through to professional qualifications. Caregiving arrangements varied from being a lone caregiver (*n* = 2) to the involvement of the extended family (*n* = 11). None had received any training in caregiving, and only one family had an arrangement with a paid caregiver.

### Interviews

All participants spoke at least one of the following languages fluently: Kannada, English, Hindi, or Urdu. Several pilot interviews were conducted by the interviewer, which led to the development of an interview guide and refining interviewing skills for exploring coercion sensitively. Participants were interviewed individually using narrative methods after obtaining informed consent. Coercion in care giving is potentially sensitive subject; all interviews were conducted in the research unit ensuring sufficient privacy and confidentiality to the participants. Narratives refer to stories made by people to understand, interpret the world around them, and create some meaning within their lives. Narrative interviewing is a technique predominantly employed to understand these stories and the lives of individuals [21]. The focus is on lived stories, expressed in the form of words or text, with minimal coaching or directing from the interviewer [22, 23]. The interviews were conducted in local languages and transcribed by the interviewers themselves (VD and MB). All recordings and transcriptions were re-examined by another researcher (MK) for accuracy.

### Analysis

The transcripts were analysed manually utilising Interpretative Phenomenological Analysis (IPA) to develop key themes. Interpretative Phenomenological Analysis (IPA) aims to explore how participants make sense of their personal and social world and has social cognition as its central analytic focus [21]. The approach is phenomenological in that it attempts to explore an individual’s personal perception, rather than produce an objective statement, of an object or event. IPA assumes a chain of connection between people’s use of language and their thinking and emotional state [21]. However, it also recognises that it is impossible to gain an insider’s perspective completely. Access depends upon and is complicated by the interpretations of the researcher [21]. The method recognises that people struggle to express what they are thinking and feeling and the researcher often has to interpret people’s mental and emotional state from what they say [22]. The onus in this method is to make those interpretations explicit and open to challenge and modification. IPA involves a two-stage process of interpretation known as a double hermeneutic: the participant trying to make sense of their world, whilst the researcher is also trying to make sense of the participant making sense of their own world [24]. IPA was conducted by the investigators (MK, VD, PK, and SJ) whilst revisiting the transcripts for accuracy and consistency raising data trustworthiness.

## Results

Caregivers reported the symptoms of physical and emotional burn-out, a lack of respite care and shared caregiving arrangements, limited knowledge of dementia, and a complete lack of community support services. Caregivers reported restrictions on their lives through not being able to participate in social activities (alone or with the PwD), reduced income and job opportunities, and restrictions on their movement that impacted on their physical and emotional well-being, with both parties feeling coerced into the situation.

## Findings

### Reasons for using coercion

The following key findings were identified as expressed intent by caregivers for applying coercive measures towards PwD. The best examples are used.

a. Coercion for safeguarding funds: Restricting access to finances to avoid mismanagement was common in those receiving pensions, particularly in those who were earlier involved in making financial decisions.

*“so, we don’t give him any money. He keeps asking for money the whole day”* (Caregiver participant 4)

Restricting physical movement both outside and inside the house was commonly employed to ensure physical safety of PwDs. The use of locked doors within the accommodation and outside perimeter confinement was adopted.

*“She’d go searching for her, trying to get her back and I told her, Mummy that is not possible”* (Caregiver participant 6)

b. Coercion for managing physical health: Restriction and enforcement of diet and medication occurred in those with physical comorbidities, as this was considered as a measure to prevent worsening of physical health.

“*Limited food we are giving as she has sugar (Diabetes). Sugar tablets should be given, and we have to see that things should not go beyond their limit…”* (Caregiver participant 10)

c. Coercion for the management of behavioral problems: When caregivers had access to medications, they tended to use these in preference to non-pharmacological management of any challenging behaviors. The administration of medication was exclusively tailored towards controlling behavioral problems by sedating PwD.

*“So she only asks about her brothers and sisters who have died long… Hhmmm…each and every time we used to call her giving the tablets tell something…”* (Caregiver participant 12)

*‘‘ the medication was not working….so we get more and then changed again……..this let us have some sleep’’ (*Caregiver participant 4)

d. Coercion resulting from routine and structural pressures

Daily structure and routine may assist those with longer term conditions to function and make the most of the situation, but may equally restrict the lives of both in the family partnership and roles become reversed. Caregiver participant 13 described a daily routine that is unforgiving and challenging both for the caregiver and the PwD.

“*By 2 o’clock she will go, by that time I have to feed her and take her to the bed and I will have one and half hours rest in the afternoon. After 5 o’clock once again many things will come. Then purchasing vegetables other things, I have to bring them prepare for the next day. By 7.30, she has to be fed, 8 o’clock she has to be taken to the bed. When she goes to the bed, I can’t leave her can I. This is the routine. How can I go to some doctor and learn these things. If somebody is there, where people are there who can relieve me”* (Caregiver participant 13).

*“I have to get to work by 8 am. The only way to keep him safe is locking in the house” *(Caregiver participant 3)

## Methods of coercion

Coercive measures imposed on family members ranged from restricting liberty through to physical, emotional, or social deprivation. Examples include:

1. Inappropriate use of psychotropic featured strongly, with most PwDs receiving them for sedation. These drugs were prescribed by doctors predominantly to control behavioral problems and caregivers reported administering doses much higher than the prescribed limit. Family members administered them to sedate PwD, to facilitate a few hours respite or an undisturbed night’s sleep for themselves.
2. Seclusion and environmental restraint: Though none of the caregivers reported using seclusion (enforced isolation) throughout 24 h, many resorted to using it as a way of dealing with behavioral problems at specific times, e.g., when they had visitors. Seclusion predominantly took the form of locking the PwD in a room or locking the main gates, or accommodation doors, so that the PwD was confined to one part of the house.
3. Restriction of dietary intake: Restricting the quantity of fluids and solid foods featured in spite of reported requests by PwD for a repeat serving of a food/fluids of their liking or choice. Fluid restriction was used to reduce the frequency of urinary incontinence (irrespective of any physical diagnosis necessity).
4. Restriction of access to finances: One or two episodes of mismanagement of bills by PwD led to the complete restriction of access to funds, and financial management was taken over by caregivers. It was also observed that such restrictions to funds commonly resulted in disturbed behavior from PwD.
5. Restriction of participation in social events: Behavioral problems, incontinence, and drooling of saliva were among the prominent reasons given for restricting PwD from participation in social events. This identifies themes of shame and stigma associated with the caregiving for a PwD.

These coercive methods adopted above by caregivers, we argue and illustrate limited knowledge, awareness, and poor coping strategies that may also compound neglect of PwD.

## Discussion

To our knowledge, this is the first study to qualitatively explore coercive practices used by caregivers towards community dwelling older adults in an LMIC setting. There are relatively few studies from high-income settings examining coercion towards to PwD, predominantly focusing on restraint in hospital and care settings [15, 25]. The existing literature both from high- and low-income settings has predominantly examined the prevalence of violence and coercive methods in hospital or long-term care setting. There is limited research even in high-income setting, exploring coercive practices by family caregivers in community setting. The findings from this study provide some evidence of coercive practices from community caregivers, and challenges encountered by them and the PwD in South India.

This study has several strengths. We interviewed the primary caregivers and thereby were able to obtain some insights into the burden of caregiving and coercive methods in settings with limited services to support PwD and their families. The strength of this study is how it illustrates themes of relevance for the unaided relatives of the family (or the extended family) caregivers in the South Indian context. This is probably relevant as a contrast between LMIC countries and high-income countries, where the former both have weaker social support systems as well as stronger family values than the high-income countries. This study is part of a much larger epidemiological study. The sample is appropriately selected and is of a sufficient size for the method deployed.

We acknowledge certain limitations. The interviews were conducted broadly to explore the experience of caregiving in a resource limited setting. Therefore, an in-depth exploration of the methods of coercion employed, and their impact, was not possible. It is likely that caregivers, out of respect to the elders, did not disclose the full extent of the caregiver burden, and equally likely that they underreported the use of coercive measures. This study investigated coercion only from the caregiver’s perspective, and this research should be complemented by interviews of PwD and direct observations of caregiving (e.g., ethnographic study). This study investigated individuals from one city in South India (Mysore), and the results may not be generalisable to other community contexts or cultures. Study findings should be interpreted in the context of these specific methodological shortcomings. Nonetheless, the study has revealed the nature of coercive measures on the caregiver and PwD, which has far reaching clinical concerns and should be routinely addressed with families caring for PwD.

Inappropriate use of psychotropics, particularly sedatives was a recurring theme and this requires further investigation of prescribing practices. Our study findings are similar to others reporting coercive practices in institutional settings from higher income countries [15, 25].

Caregivers in our study realise that they are providing sub-standard care and would like to do better but have limited choice due to economic reasons. None of the caregivers in our study were receiving any financial support from governmental or non-governmental agencies. Caregivers and family members should be trained to care for PwD in community settings. They should also be informed about available support services, including the charitable and voluntary sector (including financial help). The limited knowledge is undoubtedly a major driver for the families to employ a range of measures that are inappropriate and at times dangerous [26].

Many low- and middle-income countries, including India, do not have the resources to support the increasing health and social care demands associated with an ageing population, and have significant infrastructural barriers to accessing existing social protection schemes [27]. This has resulted in an absence of formal support or monitoring services for safeguarding vulnerable older adults with dementia. If left unchecked, both families and those persons with dementias are at risk of further social isolation and significant neglect.

There is a need for training health and social care professionals to better understand the use of coercive measures and its impact on persons with dementia. Educational support and practical assistance from mental health and community services may mitigate some of the demands faced by caregivers in the community. Caregivers, professionals, and the wider social community need awareness training in the identification and minimisation of coercion among PwD. The issues and findings from this small scale study are concerning, and highlight an urgent need for larger multicenter work to be undertaken across cultures and continents, because we suspect that they are global issues.

## Abbreviations

PwD: Person with dementia
LMIC: Low- and middle-income country
